# Cognitive Stimulation and Strength Training in Older Adults with Mild Cognitive Impairment: A Randomized Controlled Trial

**DOI:** 10.3390/diagnostics15121477

**Published:** 2025-06-10

**Authors:** Juan Miguel Muñoz-Perete, María del Mar Carcelén-Fraile, Yolanda Castellote-Caballero, María del Carmen Carcelén-Fraile

**Affiliations:** 1 Department of Health Sciences, Faculty of Health Sciences, University of Jaén, 23071 Jaén, Spain; jmmunoz@ujaen.es (J.M.M.-P.);; 2 Department of Health Sciences, Faculty of Health Sciences, University of Atlántico Medio, 35017 Las Palmas de Gran Canaria, Spain; 3 Department of Education Sciences, Faculty of Social Sciences, University of Atlántico Medio, 35017 Las Palmas de Gran Canaria, Spain

**Keywords:** mild cognitive impairment, cognitive stimulation, strength training, older adults

## Abstract

**Background/Objectives:** The global increase in life expectancy has led to a higher prevalence of cognitive and physical decline in older adults, particularly in those with mild cognitive impairment (MCI). This study aimed to evaluate the effects of a combined cognitive stimulation and resistance training intervention on cognitive performance, physical function, and fall risk in older adults with MCI. **Methods**: A randomized controlled trial was conducted with 80 community-dwelling older adults diagnosed with MCI. Participants were randomly assigned to an experimental group (EG), which received a 12-week intervention consisting of cognitive stimulation and progressive strength training, or a control group (CG), which maintained their usual routine. Pre- and post-intervention assessments included measures of cognitive function, verbal fluency, attention, processing speed, executive function, gait, balance, fall risk, and lower- and upper-body strength. **Results**: The EG showed significant improvements compared with the CG in cognitive impairment, verbal fluency, processing speed, balance, gait, and risk of falls (all *p* < 0.05), with effect sizes ranging from moderate to large. Notably, strength gains were observed in both lower body and grip strength. Attention and executive function also improved in the EG, although with smaller effect sizes. No adverse events were reported. **Conclusions**: A combined intervention of cognitive stimulation and resistance training is effective in improving multiple domains of cognitive and physical function in older adults with MCI. These findings support the integration of multidomain interventions in clinical and community settings to promote autonomy, reduce fall risk, and delay cognitive and functional decline. Future studies should explore the long-term sustainability of these effects and the individual contribution of each intervention component.

## 1. Introduction

Mild cognitive impairment (MCI) is a clinical condition that lies between normal cognitive aging and the early stages of dementia [1]. It is characterized by an observable decline in at least one cognitive function, without significant loss of functional autonomy [2]. However, this stage can represent a critical point for intervention, as a significant proportion of people with MCI progress to dementias such as Alzheimer’s disease [3]. For this reason, it is essential to identify the cognitive and functional domains that are compromised at this stage and explore strategies that preserve or improve these capacities [4].

From a cognitive perspective, people with MCI present impairments in various higher-order functions. One of the most frequently affected areas is memory, particularly episodic memory, which impacts the ability to recall recent events or acquire new information [5]. This difficulty is often the main reason for consultation in older adults. Likewise, impairments in attention and concentration are observed, which impact the ability to maintain focus on prolonged tasks or appropriately switch between stimuli [6]. Another commonly compromised domain is executive function, related to processes such as planning, decision-making, and inhibitory control; these difficulties can lead to errors in everyday activities that require reasoning or sequencing [7]. Furthermore, decreased cognitive processing speed is frequently found, which slows the execution of mental tasks and can contribute to a general feeling of sluggishness [8]. Finally, language—especially semantic verbal fluency—can also be affected, manifesting as difficulties finding appropriate words or generating lists within specific categories [9].

On a functional level, although MCI does not imply a total loss of independence, various studies have shown that signs of decline in physical and motor ability begin to appear [10]. One of the most relevant areas is general mobility, which can progressively deteriorate due to decreased coordination between the neuromuscular and cognitive systems, reflecting a decreased ability to walk safely, climb stairs, or react to obstacles [11]. Likewise, muscle strength, especially in the lower extremities, tends to decrease, negatively impacting stability and the ability to perform basic activities such as getting up from a chair or maintaining balance [12]. Dynamic and static balance can also be altered, significantly increasing the risk of falls, one of the leading causes of serious injuries in older adults [13]. This functional impairment, although incipient, can influence self-perception of health and limit participation in social activities, which in turn could accelerate cognitive decline due to lack of stimulation and mobility [14].

In this context, cognitive stimulation has proven to be an effective tool for preserving or even improving mental function in older adults with cognitive impairment [15]. Through structured activities that train memory, attention, language, and executive functions, this intervention has been observed to enhance neuroplasticity, slow the progression of impairment, and maintain autonomy in daily activities [16]. Furthermore, it has been associated with improved mood, self-esteem, and quality of life, also acting as a protective factor against social isolation and depression, common in this population [17].

Furthermore, strength training has taken on a central role in preventing functional decline associated with aging [18]. This type of exercise has been shown to not only improve muscle strength and balance but also have positive effects on cognitive function, possibly through mechanisms such as increased cerebral blood flow, the release of neurotrophic factors, and improved synaptic connectivity [19]. Furthermore, by strengthening muscles and improving stability, the risk of falls is significantly reduced and the performance of basic tasks is facilitated, thus promoting greater independence [20].

The combination of both strategies has shown even greater therapeutic potential by simultaneously addressing the mental and physical components of aging. This synergy allows for a more comprehensive intervention, which not only enhances the specific functions of each area but also fosters a virtuous cycle between physical well-being and cognitive performance [21]. Several studies suggest that the combined effects can have a greater impact on overall functionality, emotional state, and quality of life in older adults compared with interventions applied in isolation [22,23].

The combined presence of cognitive and functional impairments in people with MCI highlights the need for comprehensive interventions that address both aspects simultaneously. In this context, the combination of cognitive stimulation and strength training emerges as a promising strategy to slow the progression of decline and improve quality of life in this vulnerable population [24]. Despite the growing evidence supporting both cognitive and physical training interventions in older adults with MCI, most studies have focused on isolated interventions, conducted under controlled or laboratory settings, with limited ecological validity. There is a lack of randomized controlled trials that implement structured, combined protocols in real-world community contexts, targeting multiple functional domains beyond cognition alone. Furthermore, few studies explore the feasibility and effectiveness of such interventions in populations with early-stage cognitive decline who are still functionally autonomous. This study aims to address this gap by evaluating the effects of a combined cognitive stimulation and strength training program specifically designed for older adults with MCI living in the community.

## 2. Materials and Methods

### 2.1. Research Design and Participants

This research was carried out as a randomized controlled trial from January to April 2025. The research was approved by the Mid-Atlantic University Ethics Committee (CEI05-013) and was conducted in accordance with the principles established in the Declaration of Helsinki. It was also registered in the ClinicalTrials.gov database under identification number NCT06666088. Prior to the intervention, all participants received detailed information about the study objectives and procedures and signed written informed consent.

During the recruitment stage, 91 older adults with a diagnosis of mild cognitive impairment were approached. Following the screening, four individuals chose not to participate and two failed to meet the eligibility criteria. Consequently, 82 participants were enrolled and randomly allocated into one of two study groups: the experimental group, which underwent a combined program of cognitive stimulation and strength training, and the control group, which did not receive the intervention but was provided with general advice on physical and mental well-being.

### 2.2. Eligibility Criteria

To participate in the study, older adults had to meet the following inclusion criteria: (i) be 60 years of age or older; (ii) have a diagnosis of MCI, confirmed by neuropsychological assessment or by a healthcare professional; (iii) be physically able to participate in moderate physical activity and perform strength exercises under supervision; (iv) be willing and able to regularly attend sessions of the cognitive stimulation and physical training program; and (v) sign an informed consent form, declaring their understanding and acceptance of the study objectives and procedures.

Individuals were excluded if: (i) they had a diagnosis of moderate or severe dementia or other advanced cognitive impairment that would prevent active participation in the program; (ii) they had serious or limiting physical conditions, such as cardiac or respiratory diseases, or musculoskeletal injuries that made strength exercises difficult; (iii) suffered from psychiatric disorders, such as major depression or schizophrenia, or used substances that interfered with program compliance; (iv) were simultaneously participating in other cognitive stimulation or physical training programs; (v) were unable to understand basic instructions or lacked family or social support to attend the program if necessary; and (vi) presented uncorrected hearing or visual impairments that interfered with their ability to follow instructions or complete the intervention activities.

### 2.3. Randomization

The participants in this study were randomly assigned to either an experimental group or a control group, with an equal distribution in a 1:1 ratio. The randomization was conducted using a computer-generated table of random numbers. Group assignments were placed in sealed, opaque envelopes and managed by personnel who were not involved in any part of the study, including participant selection, intervention delivery, or data analysis. This procedure ensured allocation concealment and helped minimize the risk of bias.

**Figure 1:**
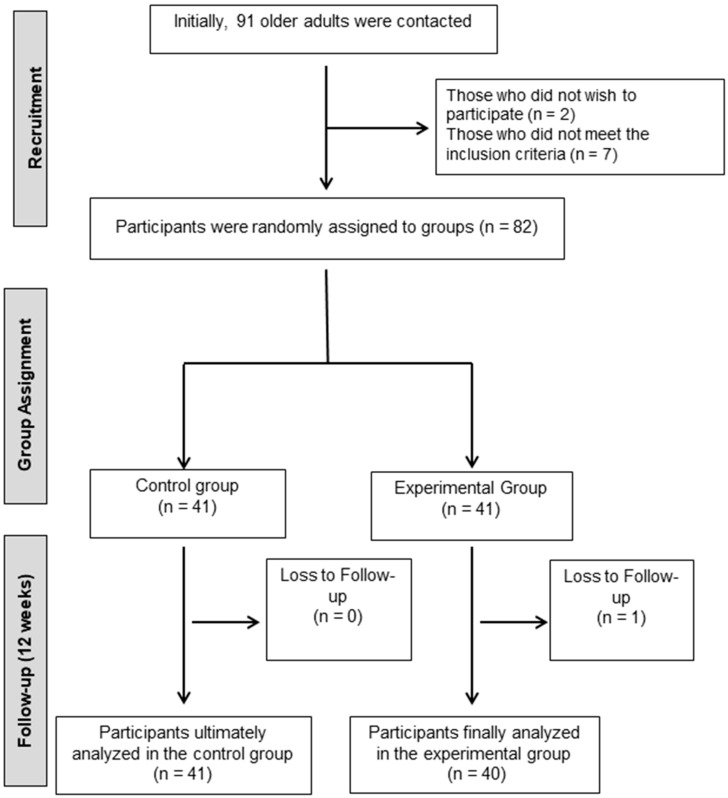
Flow diagram illustrating the flow of participants.

A total of 82 older adults were assigned to the study groups. The experimental group participated in a 12-week combined program of cognitive stimulation and strength training, structured in twice-weekly in-person sessions. The control group did not participate in the active intervention but received general recommendations on physical and mental healthcare, as well as follow-up on lifestyle habits (physical activity, sleep, nutrition, and stress levels) throughout the same period. Evaluations were conducted for both groups prior to and following the intervention (Figure 1).

### 2.4. Intervention

Participants assigned to the experimental group (EG) participated in a structured 12-week intervention program combining cognitive stimulation and strength training. Sessions were held twice weekly, lasting between 45 min and one hour per session for each program component. Activities were delivered in small groups and supervised by specialized professionals. Furthermore, in each session, the cognitive stimulation activities were performed first, followed by the physical strengthening exercises. This order was chosen to ensure optimal concentration during the cognitive tasks and allow for a more dynamic transition to the physical component of the session.

Cognitive stimulation was led by professionals in neuropsychology or occupational therapy and focused on training cognitive functions such as memory, attention, processing speed, and executive functions. Sessions included a variety of activities such as memory games, recall exercises, categorization tasks, problem-solving, and sustained attention dynamics, designed to be adapted to each participant’s level. The objective of these sessions was to maintain or improve cognitive performance through the structured stimulation of key neural networks and the promotion of brain plasticity (Table 1).

The physical component consisted of strength training aimed at improving muscle mass, balance, and coordination. The sessions were guided by a professional specializing in physical exercise for older adults. The exercises included the use of elastic bands, light weights, and body weight, focusing on large muscle groups, especially those of the lower extremities. Dynamic and static balance activities were also incorporated, progressing in intensity according to the participants’ individual abilities. The goal was to preserve functionality, prevent falls, and improve the ability to perform activities of daily living (Table 2).

The control group (CG), on the other hand, did not participate in the intervention program. However, they were provided with general information about the importance of physical and cognitive activity in healthy aging, as well as recommendations on healthy lifestyle habits. During the same twelve-week period, follow-up was conducted to document possible changes in their physical activity levels, sleep routines, diet, and stress levels. Additionally, periodic phone calls were made to remind participants not to engage in any structured cognitive or physical training programs and to report any changes in their activity routines. This procedure helped ensure adherence to the control condition. Like the experimental group, participants in the control group were assessed before and after the intervention period.

### 2.5. Outcomes

Data collection was conducted by an independent researcher, who was not involved in group assignment or intervention implementation, to ensure objectivity in the evaluation process. The data collected included sociodemographic and clinical variables relevant to sample characterization.

Comprehensive demographic and anthropometric information was obtained for each participant. Age was documented, while body weight was measured using a high-precision digital scale from Tefal, which is capable of measuring within a range of 100 g to 130 kg. Height was assessed using a calibrated stadiometer, specifically the Asimed T201 T4 model (Asimed, Istanbul, Türkiye). In addition to these physical measures, detailed sociodemographic data were collected. Marital status was categorized as married, single, separated or divorced, and widowed. Employment status was recorded as either employed, unemployed, or retired. Educational background was classified into four levels: no formal education, primary education, secondary education, and university education. This array of baseline data enabled the researchers to construct a thorough initial profile of the study sample and to explore potential associations between these characteristics and the outcome variables measured before and after the intervention.

#### 2.5.1. Cognitive Impairment

The Montreal Cognitive Assessment (MoCA) is a concise yet multidimensional screening instrument composed of 12 tasks designed to evaluate seven core cognitive domains. These include visuospatial and executive functions, assessed through activities such as tracing a geometric figure, copying a three-dimensional cube, and drawing a clock; naming abilities; attentional capacity; working memory, measured by recalling numerical sequences in reverse order; and sustained attention, evaluated through a continuous performance task. Additionally, the assessment explores language proficiency through sentence repetition and verbal fluency tasks, abstract reasoning through conceptual verbal tasks, as well as immediate and delayed memory recall and orientation in time and space. The MoCA yields a maximum score of 30 points, with scores of 26 or above being interpreted as indicative of intact cognitive functioning [25].

#### 2.5.2. Verbal Fluency

Verbal fluency was evaluated using the Isaac test, a standardized measure in which participants were instructed to generate as many words as possible that belonged to a given semantic category—such as animals, fruits, cities, or colors—within a time frame of 60 s. Each semantic category permitted a maximum of 10 points, leading to a cumulative total score of 40. Higher scores on this assessment are interpreted as indicative of superior verbal fluency and lexical retrieval capacity [26].

#### 2.5.3. Executive Functions

Executive functioning was measured using the Trail Making Test (TMT), a neuropsychological instrument designed to evaluate cognitive processes involving motor coordination and visual tracking within time-constrained conditions. The assessment comprises two distinct components. The first component, known as TMT A, requires individuals to connect a sequence of numbered circles in ascending order, primarily evaluating sustained attention and processing speed. The second component, TMT B, demands the alternation between numbers and letters in sequential order, thereby placing greater demands on cognitive flexibility and executive control. Performance on this test is quantified based on the time taken to complete each part, with longer completion times being interpreted as indicative of reduced cognitive efficiency [27].

#### 2.5.4. Processing Speed

The Digit Symbol Substitution Test (DSST) [28] is a paper-based cognitive assessment administered on a single page. It requires participants to accurately pair symbols with their corresponding numbers using a reference key positioned at the top of the sheet. The task involves reproducing the appropriate symbol in the designated space beneath each number in a sequential row. Scoring is determined by the total number of correct symbol substitutions completed by the participant within a time limit of 90 s, reflecting processing speed, attention, and visuomotor coordination.

#### 2.5.5. Balance, Gait, and Falls of Risk

The Tinetti Scale was employed to evaluate physical parameters related to balance, gait, and the associated risk of falling [29,30]. This instrument is composed of two distinct sections. The first section, consisting of nine items, assesses both static and dynamic balance and yields a maximum score of 17 points. The second section evaluates gait through seven items, with a maximum possible score of 12 points. The cumulative score from both sections provides an overall indication of fall risk, where higher total scores correspond to greater stability and a lower likelihood of falling. Specifically, total scores ranging from 19 to 24 suggest a moderate risk of falls, while scores below 19 are interpreted as indicative of a higher fall risk.

#### 2.5.6. Grip Strength

A dynamometer [31] was employed to record the number of arm curl repetitions performed within a thirty-second interval for both the right and left arms, each using a dumbbell. Participants began the assessment seated on a standard chair with a seat height of forty-three centimeters, keeping one arm fully extended at their side. The movement sequence required a gradual upward rotation of the palm while flexing the elbow through its entire range of motion, followed by a complete extension to return to the initial position. In order for each repetition to be considered valid, the participant was required to execute full flexion and extension of the elbow, ensuring a complete range of movement.

#### 2.5.7. Lower Body Strength

The Chair Stand Test [32] is a functional assessment designed to evaluate the strength of the lower extremities by measuring an individual’s capacity to repeatedly transition from a seated to a standing position. The procedure begins with the participant seated on a standard chair, feet positioned flat on the floor, and arms crossed firmly over the chest. Upon receiving the signal to begin, the individual is instructed to stand up and sit back down as many times as possible over a thirty-second period. The final score corresponds to the total number of correctly executed repetitions, with a higher count reflecting superior muscular strength in the lower limbs. This test is widely utilized in geriatric populations to evaluate balance and physical functionality, given the pivotal role of leg strength in carrying out essential daily movements such as rising from a chair or ambulating independently.

### 2.6. Sample Size Calculation

The sample size was calculated using G*Power software (version 3.1), considering a mixed analysis of variance design with two groups (experimental and control) and two measurements (pre- and post-intervention). A medium effect size (f = 0.25), a significance level of α = 0.05, and a statistical power (1 − β) of 0.80 were considered. This effect size was selected based on previous studies involving multicomponent interventions targeting cognitive function in older adults with mild cognitive impairment. For instance, Suzuki et al. [33] employed the same effect size in a randomized controlled trial assessing the impact of combined physical and cognitive training on cognition. Under these parameters, the minimum sample size required was 82 participants in total (41 per group) to detect significant effects in the interaction between group and time. Therefore, the sample used in the study meets the statistical requirements necessary to guarantee the validity of the analyses performed.

### 2.7. Statistical Analysis

For data analysis, means and standard deviations were calculated for each of the variables included in the study. To compare the results between the two groups (experimental and control), Student’s *t* test was applied for independent samples. In addition, a repeated measures analysis of variance (mixed ANOVA) model was used, considering the group (EG vs. CG) as a between-groups factor and the assessment time (before and after the intervention) as a within-subjects factor. Each dependent variable was analyzed independently, including those related to cognitive status, verbal fluency, executive function, and functional status. The interaction between group assignment and measurement time was specifically examined to identify potential differential effects attributable to the intervention. To estimate the magnitude of the differences between groups, the effect size was calculated using Cohen’s d, considering values of ≤0.2 as small effect, 0.5 as moderate effect, and ≥0.8 as large effect [34]. A statistical significance level of *p* < 0.05 was set for all comparisons. The analyses were performed using SPSS statistical software, version 17.0 (SPSS Inc., Chicago, IL, USA).

## 3. Results

In this study, 30.90% of the participants were men and 69.10% were women. The mean age of the participants was 71.17 ± 4.73 years. The majority of participants were retired (76.50%), married (23.70%), and had completed secondary education (43.20%) (Table 3). When comparing the groups, no significant differences were observed in any of the sociodemographic characteristics.

The experimental group (EG) showed a statistically significant improvement in cognitive impairment scores after the intervention (pre: 21.45 ± 1.11; post: 22.30 ± 1.22; t(39) = −3.189, *p* = 0.003, d = 0.73), indicating a moderate effect size. In contrast, the control group (CG) showed a decline (post: 20.95 ± 1.14), resulting in a significant between-groups difference at post-test (t(79) = −5.136, *p* = 0.000, d = 1.14), with a large effect size. The mixed ANOVA confirmed a significant Group × Time interaction (F(1,79) = 15.170, *p* = 0.000, η^2^ = 0.161), as well as a significant main effect of Group (F(1,79) = 13.183, *p* = 0.000, η^2^ = 0.143), but no significant main effect of Time (F(1,79) = 1.542, *p* = 0.218, η^2^ = 0.019), as shown in Table 4. These findings indicate that the intervention produced meaningful cognitive improvements that exceeded natural variation over time and reflect real benefits attributable to the program.

Following the intervention, the experimental group (EG) showed a significant improvement in verbal fluency scores (pre: 26.95 ± 2.61; post: 28.48 ± 2.40), with a large effect size (t(39) = −3.935, *p* = 0.000, d = 0.61). In contrast, the control group (CG) did not show a statistically significant change (pre: 26.83 ± 2.73; post: 26.29 ± 2.94; t(40) = −1.294, *p* = 0.202). The mixed ANOVA confirmed a significant Group × Time interaction (F(1,79) = 12.093, *p* = 0.001, η^2^ = 0.133) and a post-intervention between-groups difference (t(79) = −3.654, *p* = 0.000, d = 0.82), indicating a large and meaningful treatment effect. This suggests that the combined cognitive and physical intervention was effective in enhancing verbal fluency, which reflects improvements in semantic memory access and executive function—key domains often affected in individuals with MCI. After the intervention, the experimental group (EG) exhibited a significant improvement in attention and processing speed (t(39) = 9.973, *p* = 0.000, d = 1.02), reflecting a large effect size. The between-groups comparison at the post-test revealed a statistically significant difference (t(79) = 2.173, *p* = 0.033, d = 0.48), with a small-to-moderate effect size. A mixed ANOVA confirmed a significant Group × Time interaction (F(1,79) = 89.542, *p* = 0.000, η^2^ = 0.531), as well as a significant main effect of Time (F(1,79) = 73.992, *p* = 0.000, η^2^ = 0.483), indicating that the observed changes over time were mostly attributable to the intervention. No significant main effect was observed for Group (F(1,79) = 0.040, *p* = 0.843, η^2^ = 0.001), as shown in Table 4. These results suggest that the intervention effectively enhanced processing speed and sustained attention—two cognitive functions essential for daily functioning and frequently impaired in individuals with MCI.

In the domain of executive function, statistical analyses did not reveal a significant Group × Time interaction (F(1,79) = 3.387, *p* = 0.069, η^2^ = 0.041), nor a significant main effect of Time (F(1,79) = 3.880, *p* = 0.052, η^2^ = 0.047). However, a significant main effect of Group was found (F(1,79) = 4.586, *p* = 0.035, η^2^ = 0.055), as reported in Table 4. These findings suggest that, although there was no statistically significant differential change over time between groups, the experimental group exhibited overall higher executive functioning compared with the control group. Further studies with larger samples or longer intervention periods may help clarify whether this trend reflects a true treatment effect or individual baseline variability.

The experimental group (EG) showed a statistically significant improvement in processing speed following the intervention (pre: 51.03 ± 6.74; post: 60.13 ± 16.39), with a within-groups comparison confirming this change (t(39) = −16.839, *p* = 0.000, d = 0.50), indicating a small but meaningful effect size. A post-test comparison also revealed a significant difference between the EG and the control group (CG: 55.68 ± 11.16), with a small between-groups effect size (t(79) = −5.136, *p* = 0.000, d = 0.31). The mixed ANOVA showed a significant Group × Time interaction (F(1,79) = 50.099, *p* = 0.000, η^2^ = 0.388) and a significant main effect of Time (F(1,79) = 39.990, *p* = 0.000, η^2^ = 0.336), but no significant main effect of Group (F(1,79) = 0.050, *p* = 0.824, η^2^ = 0.001), as presented in Table 2. These results suggest that the observed gains in processing speed were primarily driven by the intervention, reinforcing its potential to enhance basic cognitive operations critical for task efficiency and daily functioning in older adults with MCI.

After the intervention, the experimental group (EG) showed a significant improvement in balance scores (pre: 11.12 ± 2.81; post: 12.60 ± 2.94), with a within-groups comparison revealing a significant change (t(39) = −3.364, *p* = 0.002, d = 0.51), representing a small effect size. A post-test comparison also revealed a significant difference between the EG and the control group (CG: 9.98 ± 2.54), with a large effect size (t(79) = −4.304, *p* = 0.000, d = 0.95). The mixed ANOVA confirmed a significant Group × Time interaction (F(1,79) = 9.076, *p* = 0.003, η^2^ = 0.103) and a significant main effect of Group (F(1,79) = 10.335, *p* = 0.002, η^2^ = 0.116), although the main effect of Time did not reach significance (F(1,79) = 2.472, *p* = 0.120, η^2^ = 0.030), as shown in Table 2. These results indicate that the intervention had a meaningful impact on balance performance, which is a critical factor in fall prevention and mobility preservation among older adults with MCI.

The experimental group (EG) showed a significant improvement in gait following the intervention (t(39) = −2.189, *p* = 0.035, d = 0.36), indicating a small effect size. Post-test comparisons revealed a significant difference between the EG and the control group, with a large effect size (t(79) = −4.211, *p* = 0.000, d = 0.94). The mixed ANOVA confirmed a significant Group × Time interaction (F(1,79) = 5.129, *p* = 0.026, η^2^ = 0.061) and a significant main effect of Group (F(1,79) = 12.705, *p* = 0.001, η^2^ = 0.139), while the main effect of Time was not statistically significant (F(1,79) = 0.340, *p* = 0.561, η^2^ = 0.004), as reported in Table 4. These findings suggest that the intervention contributed to improvements in gait, a key component of functional mobility and a predictor of fall risk in older adults with MCI.

The experimental group (EG) experienced a statistically significant improvement in fall risk scores after the intervention (t(39) = −3.466, *p* = 0.001, d = 0.49), indicating a small effect size. A post-test comparison also revealed a significant difference between the EG and the control group, with a large effect size (t(79) = −5.346, *p* = 0.000, d = 1.18). The mixed ANOVA confirmed a significant Group × Time interaction (F(1,79) = 13.433, *p* = 0.000, η^2^ = 0.145), as well as a significant main effect of Group (F(1,79) = 15.333, *p* = 0.000, η^2^ = 0.163), while the main effect of Time was not significant (F(1,79) = 2.215, *p* = 0.141, η^2^ = 0.027), as shown in Table 2. These findings suggest that the intervention was effective in reducing fall risk among older adults with MCI—an outcome of high clinical relevance given its association with morbidity, loss of independence, and institutionalization.

The experimental group (EG) showed a significant improvement in lower body strength following the intervention (t(39) = −2.275, *p* = 0.026, d = 0.30), indicating a small effect size. A post-test comparison between the EG and the control group also revealed a significant difference, with a small-to-moderate effect size (t(79) = −2.275, *p* = 0.026, d = 0.50). The mixed ANOVA revealed a significant Group × Time interaction (F(1,79) = 24.740, *p* = 0.000, η^2^ = 0.239) and a significant main effect of Time (F(1,79) = 20.651, *p* = 0.000, η^2^ = 0.207), but no significant main effect of Group (F(1,79) = 0.159, *p* = 0.691, η^2^ = 0.002), as reported in Table 2. These findings indicate that the intervention was effective in improving lower limb strength—an essential component for mobility, independence, and fall prevention in older adults with MCI.

The experimental group (EG) showed a significant improvement in grip strength following the intervention (t(39) = −3.147, *p* = 0.003, d = 0.29), indicating a small effect size. A post-test comparison revealed a statistically significant difference between the EG and the control group, with a large effect size (t(79) = −3.498, *p* = 0.001, d = 0.78). The mixed ANOVA confirmed a significant Group × Time interaction (F(1,79) = 10.075, *p* = 0.002, η^2^ = 0.113) as well as a significant main effect of Group (F(1,79) = 6.197, *p* = 0.015, η^2^ = 0.073), while no significant main effect of Time was observed (F(1,79) = 0.094, *p* = 0.761, η^2^ = 0.001), as presented in Table 2. These results indicate that the intervention contributed to an improvement in upper limb strength, which plays an important role in functional independence and daily tasks such as carrying objects, rising from a chair, or using mobility aids.

## 4. Discussion

The primary objective of this study was to evaluate the effects of a combined intervention involving cognitive stimulation and strength training on various cognitive and physical functions in older adults with mild cognitive impairment. The results revealed significant improvements in several areas, including global cognition, verbal fluency, attention and processing speed, lower and upper body strength, balance, gait, and risk of falls, suggesting that multidomain interventions can offer comprehensive benefits in this population.

Verbal fluency, a skill related to efficient word retrieval and closely linked to executive functions and semantic memory, tends to progressively deteriorate with age [35]. This deterioration is associated with structural and functional changes in the prefrontal cortex, which directly impacts spontaneous language ability [36]. In our study, EG showed significant improvements in this domain. This likely reflects the contribution of cognitive tasks involving semantic categorization and phonological retrieval, combined with the effects of exercise on brain plasticity and blood flow. These findings are consistent with other authors such as Calatayud et al. [37], who applied a structured cognitive stimulation program in older adults, finding significant improvements in verbal fluency, and Welford et al. [38], who provided evidence on yoga as an aerobic exercise. Therefore, improving verbal fluency may improve communication, social interaction, and mental flexibility in older adults with MCI, positively influencing quality of life and reducing social isolation.

Sustained attention and processing speed are cognitive functions that often deteriorate with age, affecting older adults’ ability to efficiently perform everyday tasks [39]. This deterioration is associated with structural and functional changes in brain areas such as the prefrontal cortex and parietal lobe [40]. In the present study, although the EG initially showed higher scores than the CG in these domains, the post-intervention results did not reflect the same pattern of improvement observed in other variables. One possible explanation may lie in factors such as participant fatigue, reduced motivation during the post-test phase, or the influence of test habituation (inverse learning effect), which are known to affect attention-based tasks in older populations. These aspects should be considered when interpreting the results. Nonetheless, the existing literature supports that combined interventions may be beneficial in preserving these functions. For example, Arokiaraj et al. [41] conducted a systematic review on the impact of computerized cognitive training in healthy older adults, finding significant improvements in attention and processing speed. The authors highlighted that interventions that include specific tasks for these cognitive functions can be effective in improving cognitive performance in this population. Furthermore, the study by Nishiguchi et al. [42] evaluated the effects of a television-based cognitive training program in older adults, observing improvements in processing speed and selective attention. This type of intervention, which combines recreational and technological elements, can be a useful tool for maintaining and improving cognitive functions in old age. On the other hand, physical activity has also been shown to have positive effects on attention and processing speed. According to the review by Crespillo-Jurado et al. [43], regular physical exercise in older adults is associated with improvements in various cognitive functions, including attention and processing speed. The authors suggest that exercise may promote neuroplastic changes in the brain, thereby improving cognitive performance.

Balance is an essential motor function that tends to deteriorate with age due to multiple factors, such as loss of muscle strength, decreased proprioception, and impaired neuromuscular coordination [44]. In our study, The EG showed significant improvements in balance. This may be explained by enhanced proprioception and neuromuscular control through resistance training, along with improved postural integration via cognitive engagement during physical tasks [45,46]. Our results align with other studies, showing that dual-task interventions benefit postural control in older adults. However, balance was assessed using clinical tests that may not fully capture subtle postural changes; future research should include objective measures such as force platforms. Given the close relationship between balance and fall risk, these results support the integration of cognitive-motor training in fall prevention programs, particularly in institutional or frail populations.

Gait is a complex motor function that tends to deteriorate with age, becoming slower and more unsteady due to loss of muscle strength, decreased proprioception, and impaired neuromuscular coordination [47]. In our study, the EG showed significant improvements in gait compared with the CG. These findings are consistent with recent studies highlighting the efficacy of combined interventions for improving gait in older adults, such as Nascimento et al. [48], who conducted a pilot study in older women participating in a 12-week physical–cognitive training program, and Appeadu et al. [49], who evaluated the effects of an interactive cognitive–motor training program in older adults over 24 weeks. Improved gait supports independence in instrumental activities of daily living (IADLs), such as shopping, community mobility, or visiting a healthcare provider—critical domains for aging in place.

The risk of falls increases with age due to factors such as loss of muscle strength, imbalance, and decreased reaction time [50]. A significant reduction in fall risk was observed in the EG, most likely due to gains in strength, balance, and attention. These findings are congruent with prior meta-analyses indicating that multimodal programs are particularly effective when they include a balance challenge and are delivered at sufficient volume [51,52]. The added cognitive component may further enhance fall-related self-efficacy and awareness of environmental hazards. Nevertheless, fall risk was inferred from surrogate measures, and actual fall incidence was not tracked. These results highlight the clinical relevance of implementing combined physical–cognitive programs in both preventive and rehabilitative contexts to reduce fall risk in high-risk older adults.

The loss of lower body strength is a major contributor to functional disability in older adults, limiting essential activities such as standing up, climbing stairs, or walking. In our study, the EG showed significant improvements in lower limb strength compared with the CG, supporting the effectiveness of combining strength training with cognitive stimulation. These results are in line with previous research showing that resistance training improves muscle strength and functionality in older adults [53]. Notably, resistance training also has positive effects on cognitive health, which may enhance motivation and adherence to the intervention [54]. Therefore, incorporating cognitive elements into physical training not only supports physical capacity but also facilitates long-term engagement in older populations, making this approach especially relevant in clinical and community-based prevention or rehabilitation programs.

Handgrip strength is a key indicator of health, independence, and longevity in older adults [55]. In our study, the experimental group (EG) showed significant improvements in grip strength compared with the control group (CG), suggesting that the combination of resistance training and cognitive stimulation may be effective in mitigating this decline. These results are consistent with previous studies, such as the meta-analysis by Liao et al. [56], which demonstrated that resistance training significantly improves grip strength in older adults with sarcopenia. Similarly, Kirk et al. [57] found that machine-based resistance training enhances both functional capacity and muscle strength in this population. Additionally, incorporating cognitive stimulation into physical training may further enhance outcomes by improving cognitive health, as noted by Vieira et al. [53], which in turn could increase motivation and adherence. These findings underscore the relevance of integrated cognitive–motor interventions to maintain upper limb strength, support independence in daily activities, and reduce the risk of functional decline in older adults.

The interpretation of the results derived from this study must take into account several limitations that may influence the generalizability and robustness of the findings. First, the sample size, although adequate for detecting moderate effects, could limit the generalizability of the results to other populations of older adults, especially those with more severe clinical conditions or those who are institutionalized. Second, the duration of the intervention, although sufficient for observing significant changes in several variables, may have been insufficient for detecting long-term effects or effects in more complex cognitive functions, such as executive function. Finally, another important limitation is the lack of post-intervention follow-up, which prevents us from determining the durability of the observed effects.

## 5. Conclusions

The results of this study demonstrate that a combined intervention of cognitive stimulation and strength training can generate significant improvements in both cognitive and physical variables in older adults with mild cognitive impairment. In particular, benefits were observed in functions such as verbal fluency, processing speed, balance, gait, and muscle strength, as well as a reduction in the risk of falls. These improvements reinforce the value of multidimensional approaches that simultaneously address the physical and cognitive decline associated with aging. From a clinical perspective, the findings underscore the importance of integrating cognitive stimulation and physical exercise programs into geriatric care services and the prevention of functional decline. This intervention is not only effective but also feasible for implementation in community settings, day centers, or residential institutions, helping to maintain autonomy, prevent falls, and improve the quality of life of older adults.

## Figures and Tables

**Table 1:** Cognitive stimulation program.

Week	Main Objectives	Activities	Cognitive Functions Targeted	Materials Used
1–2	Activate basic functions	Visual memory games, shape and color recognition	Sustained attention, working memory	Flashcards, images, whiteboards
3–4	Reinforce orientation and language	Categorization games, word completion, temporal-spatial orientation tasks	Language, orientation, verbal fluency	Workbooks, teaching clock
5–6	Stimulate executive functions	Task sequencing, problem-solving in daily contexts	Planning, reasoning	Problem cards, everyday items
7–8	Strengthen episodic memory	List recall, event recall, paired associations	Short- and long-term memory	Lists, image/sound flashcards
9–10	Improve processing speed	Timed symbol–number matching, pattern identification	Processing speed, cognitive flexibility	Timer, worksheets
11–12	Consolidation and integration	Group games combining previous tasks	Cognitive impairment, social interaction	Mixed materials, interactive games

**Table 2:** Strength training program.

Week	Physical Objectives	Main Exercises	Materials Used	Progression
1–2	General activation, introduce movement	March in place, assisted squats, sit to stand	Chairs, mats	Low intensity
3–4	Strengthen lower limbs	Leg raises, unsupported squats, step-ups	Low steps, resistance bands	Increased repetitions
5–6	Add upper body strength	Bicep curls, tricep extensions with bands	Bands, 1 kg dumbbells	Increased resistance
7–8	Introduce dynamic balance	Straight-line walking, single-leg stance, body shifting	Balance beam, cones	Longer hold times
9–10	Functional integration	Mobility circuits, lifting and carrying objects	Household items, timer	Requires coordination
11–12	Consolidation	Combined strength + balance circuits	Mixed materials	Moderate and sustained activity

**Table 3:** Pre-intervention sociodemographic and clinical characteristics of the participants overall and by group.

		Total (n = 81)	Experimental (n = 40)	Control (n = 41)	*p* Value
Age		71.17 ± 4.73	70.90 ± 4.24	71.44 ± 5.21	0.092
Sex	Male	25 (30.90)	12 (48.00)	13 (52.00)	0.743
	Female	56 (69.10)	28 (50.00)	28 (50.00)	
Occupational status	Retired	62 (76.50)	30 (48.40)	32 (51.60)	0.285
	Worker	7 (8.60)	3 (42.90)	4 (57.10)	
	Unemployed	12 (14.80)	7 (58.30)	5 (41.70)	
Marital Status	Married	32 (39.50)	21 (65.60)	11 (34.40)	0.191
	Divorced	14 (17.30)	3 (21.40)	11 (78.60)	
	Single	17 (21.00)	8 (47.10)	9 (52.90)	
	Widowed	18 (22.20)	8 (44.40)	10 (55.60)	
Educational Status	Primary Education	24 (29.60)	13 (54.20)	11 (45.80)	0.776
	Secondary Education	35 (43.20)	17 (48.60)	18 (51.40)	
	University studies	22 (27.20)	10 (45.50)	12 (54.50)	
Height		71.80 ± 8.35	70.35 ± 7.97	73.21 ± 8.57	0.356
Weight		1.73 ± 0.07	1.72 ± 0.08	1.73 ± 0.07	0.492
BMI		24.03 ± 1.25	23.79 ± 1.29	24.25 ± 1.19	0.746
Cognitive impairment		21.42 ± 1.11	21.45 ± 1.11	21.39 ± 1.12	0.885
Verbal fluency		26.89 ± 2.66	26.95 ± 2.61	26.83 ± 2.73	0.758
Attention and speed		103.49 ± 40.88	113.50 ± 39.07	93.88 ± 10.78	0.988
Executive functions		184.36 ± 80.33	204.53 ± 75.43	164.68 ± 80.97	0.935
Processing Speed		53.64 ± 7.05	51.03 ± 6.74	56.20 ± 6.44	0.950
Balance		10.78 ± 2.73	11.12 ± 2.81	10.44 ± 2.63	0.911
Gait		8.89 ± 2.77	9.43 ± 2.77	8.37 ± 2.69	0.770
Risk of falls		19.67 ± 4.71	20.55 ± 5.22	18.80 ± 4.02	0.119
Grip strength		16.92 ± 3.69	17.38 ± 3.70	16.48 ± 3.67	0.919
Lower body strength		11.83 ± 3.32	11.25 ± 3.54	13.39 ± 3.03	0.257

Note: Data are presented as the mean ± standard deviation for continuous variables and as the number (percentage) for categorical variables.

**Table 4:** Effects of physical exercise and the abacus on cognitive capacities.

	EG (n = 40)		CG (n = 41)		Group			Time			Group × Time		
	Pre	Post	Pre	Post	F(80)	*p*-Value	η^2^	F(80)	*p*-Value	η^2^	F(80)	*p*-Value	η^2^
Cognitive impairment	21.45 ± 1.11	22.30 ± 1.22	21.39 ± 1.12	20.95 ± 1.14	13.183	0.000	0.143	1.542	0.218	0.019	15.170	0.000	0.161
Verbal fluency	26.95 ± 2.61	28.48 ± 2.40	26.83 ± 2.73	26.29 ± 2.94	4.972	0.029	0.059	2.780	0.099	0.034	12.093	0.001	0.133
Attention and speed	113.50 ± 39.07	79.23 ± 27.33	93.88 ± 40.78	95.51 ± 38.98	0.040	0.843	0.001	73.922	0.000	0.531	73.922	0.000	0.483
Executive Functions	204.53 ± 75.43	186.58 ± 61.78	164.68 ± 80.97	164.07 ± 53.82	4.586	0.035	0.055	3.880	0.052	0.047	3.387	0.069	0.041
Processing Speed	51.03 ± 6.74	60.13 ± 6.39	56.20 ± 6.44	55.68 ± 11.16	0.050	0.824	0.001	39.990	0.000	0.336	50.099	0.000	0.388
Balance	11.12 ± 2.81	12.60 ± 2.94	10.44 ± 2.63	9.98 ± 2.54	10.335	0.002	0.116	2.472	0.120	0.030	9.076	0.003	0.103
Gait	9.43 ± 2.77	10.38 ± 2.53	8.37 ± 2.69	7.80 ± 2.94	12.705	0.001	0.139	0.340	0.561	0.004	5.129	0.026	0.061
Risk of falls	20.55 ± 5.22	22.98 ± 4.65	18.80 ± 4.02	17.78 ± 4.08	15.333	0.000	0.163	2.215	0.141	0.027	13.433	0.000	0.145
Grip strength	17.38 ± 3.70	18.46 ± 3.79	16.48 ± 3.67	15.58 ± 3.63	6.197	0.015	0.073	0.094	0.761	0.001	10.075	0.002	0.113
Lower body strength	11.25 ± 3.54	13.95 ± 3.40	12.39 ± 3.03	12.27 ± 3.25	0.159	0.691	0.002	20.651	0.00	0.207	24.743	0.000	0.239

Quantitative variables are presented as the mean and standard deviation. EG: Experimental group. CG: Control group.

## Data Availability

The datasets generated and analyzed during the course of this study can be obtained upon reasonable request directed to the corresponding author. Public access to the data has been restricted in order to uphold the confidentiality commitments made to participants, as the study involved sensitive content. Participants were explicitly assured that their raw data would remain confidential and would not be disclosed to external parties.

## Abbreviations

The following abbreviations are used in this manuscript:

MCI	Mild cognitive impairment
CG	Control group
EG	Experimental group
MMSE	Mini-Mental State Examination
MoCA	The Montreal Cognitive Assessment
TMT	The Trail Making Test
DSST	The Digit Symbol Substitution Test
CST	The Chair Stand Test
RBT	Balance training
RT	Resistance training
V-TIME	Treadmill training program combined with virtual reality
